# Supplementary material for: The WalRK two-component system in Streptococcus pneumoniae ensures robustness of secondary wall polymer attachment
Source: mBio. 2026 Jul 22;17(8):e01075-26. doi: 10.1128/mbio.01075-26 (PMC13463712; doi:10.1128/mbio.01075-26)
Supplement: Supplementary file 1 [file mbio.01075-26-s0006.pdf]

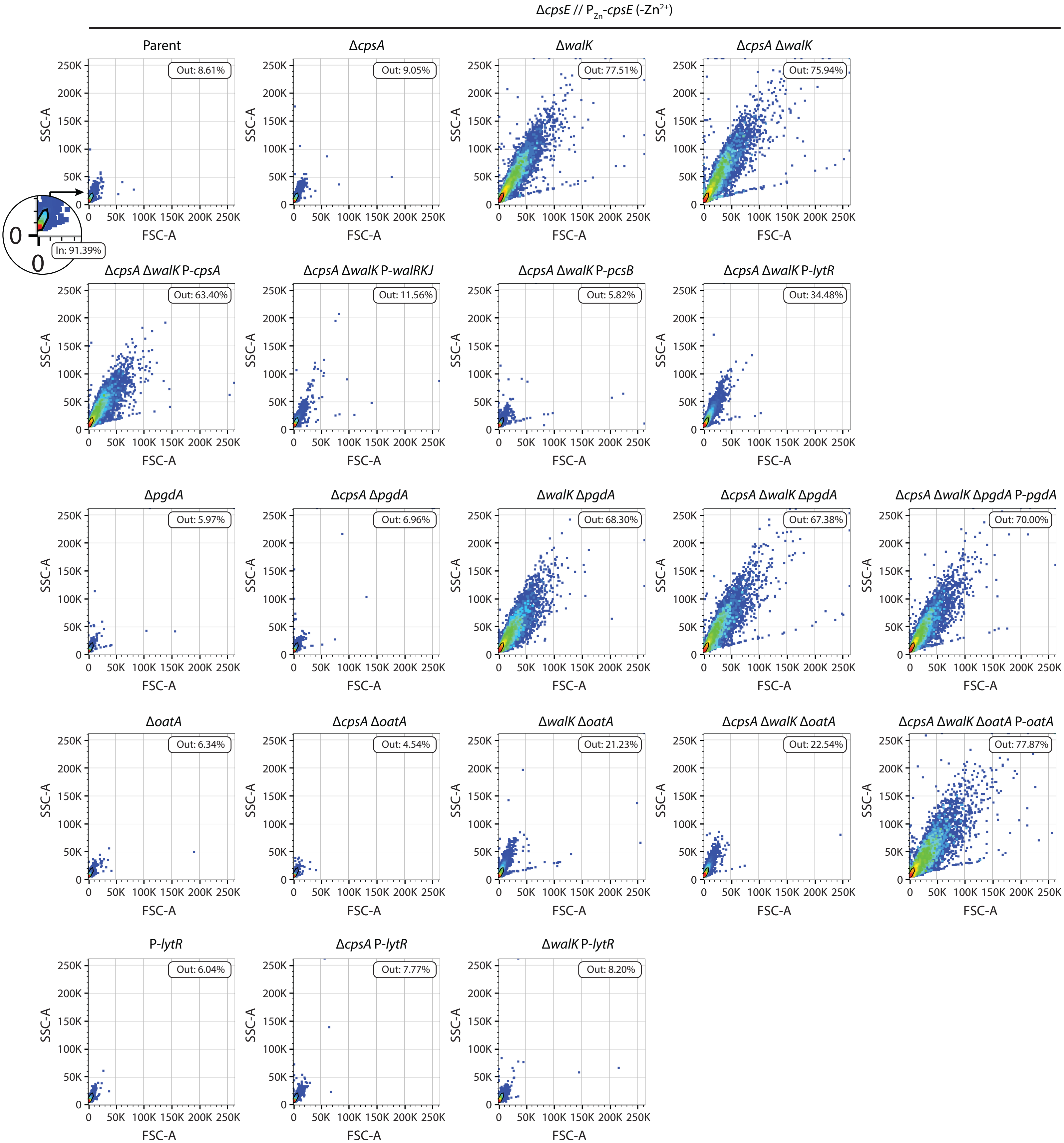
ΔcpsE // P_Zn-cpsE (-Zn2+)
Parent
Out: 8.61%
In: 91.39%
ΔcpsA
Out: 9.05%
ΔwalK
Out: 77.51%
ΔcpsA ΔwalK
Out: 75.94%
ΔcpsA ΔwalK P-cpsA
Out: 63.40%
ΔcpsA ΔwalK P-walRKJ
Out: 11.56%
ΔcpsA ΔwalK P-pcsB
Out: 5.82%
ΔcpsA ΔwalK P-lytR
Out: 34.48%
ΔpgdA
Out: 5.97%
ΔcpsA ΔpgdA
Out: 6.96%
ΔwalK ΔpgdA
Out: 68.30%
ΔcpsA ΔwalK ΔpgdA
Out: 67.38%
ΔcpsA ΔwalK ΔpgdA P-pgdA
Out: 70.00%
ΔoatA
Out: 6.34%
ΔcpsA ΔoatA
Out: 4.54%
ΔwalK ΔoatA
Out: 21.23%
ΔcpsA ΔwalK ΔoatA
Out: 22.54%
ΔcpsA ΔwalK ΔoatA P-oatA
Out: 77.87%
P-lytR
Out: 6.04%
ΔcpsA P-lytR
Out: 7.77%
ΔwalK P-lytR
Out: 8.20%
SSC-A
FSC-A
0
50K
100K
150K
200K
250K
