# Supplementary material for: The WalRK two-component system in Streptococcus pneumoniae ensures robustness of secondary wall polymer attachment
Source: mBio. 2026 Jul 22;17(8):e01075-26. doi: 10.1128/mbio.01075-26 (PMC13463712; doi:10.1128/mbio.01075-26)
Supplement: Supplementary file 2 [file mbio.01075-26-s0008.pdf]

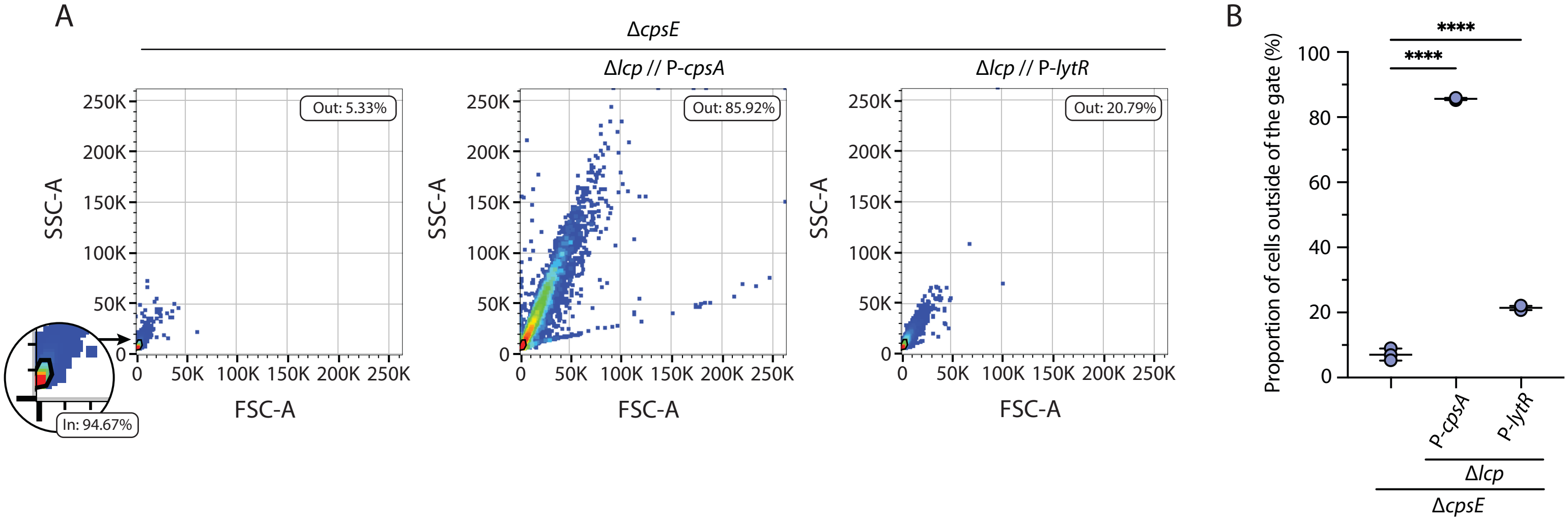

A
ΔcpsE
Δlcp // P-cpsA
Δlcp // P-lytR
Out: 5.33%
Out: 85.92%
Out: 20.79%
In: 94.67%
SSC-A
FSC-A
0
50K
100K
150K
200K
250K
B
Proportion of cells outside of the gate (%)
****
****
0
20
40
60
80
100
P-cpsA
P-lytR
Δlcp
ΔcpsE
