# Supplementary material for: The WalRK two-component system in Streptococcus pneumoniae ensures robustness of secondary wall polymer attachment
Source: mBio. 2026 Jul 22;17(8):e01075-26. doi: 10.1128/mbio.01075-26 (PMC13463712; doi:10.1128/mbio.01075-26)
Supplement: Supplementary file 3 [file mbio.01075-26-s0009.docx]

**SUPPLEMENTAL FIGURE LEGENDS**

**Fig. S1.** **Genetic interaction profiles in strains lacking *cpsD* or *cpsB*.** RB Tn-seq uncovered genetic interactions of *cpsD* (**A**) and *cpsB* (**B**). Strains NUS0680 [∆*cpsD* ∆*cpsE* // P_Zn_-*cpsE*] and NUS0731 [∆*cpsB* ∆*cpsE* // P_Zn_-*cpsE*] were grown in BHI without added Zn^2+^ at 37°C in 5% CO_2_. Cells were transformed with genomic DNA purified from a barcoded Tn library, and plated on blood agar supplemented with 0.45 mM Zn^2+^ and incubated overnight. Cells were scraped and pooled together. DNA was extracted, and the barcodes were amplified and sequenced. Points represent the average log_2_ fold change in abundance of strains with insertions in the indicated gene. Green dots highlighted the genes of interest as shown in **Fig. 3**.

**Fig. S2. Cells lacking *cpsA* and *walK* do not display widespread loss of viability in BHI.** Strain NUS0267 [∆*cpsE* // P_Zn_-*cpsE*] and its derivatives lacking *cpsA* (NUS0796), *walK* (NUS3688), or both (NUS3698) were grown in BHI at 37°C in 5% CO_2_ without added Zn^2+^. They were diluted in BHI with or without Zn^2+^. After five doublings, cells were collected, stained with the LIVE/DEAD BacLight Bacterial Viability Kit (Molecular Probes), and visualized by phase-contrast and fluorescence microscopy. Live cells stained with SYTO 9 appear green, whereas dead cells stained with propidium iodide appear red. Images were processed using Fiji. The brightness and contrast of each channel were adjusted to facilitate comparison of the two populations.

**Fig. S3. Growth defects in the ∆*cpsA* ∆*walK* mutant are caused by a mechanism unrelated to C55-P sequestration.** Strain NUS0267 [∆*cpsE* // P_Zn_-*cpsE*] and its derivatives with ∆*cpsA* (NUS0796), ∆*walK* (NUS3688), ∆*cpsA ∆walK* (NUS3698), ∆*ispA* (NUS3681), and ∆*cpsA* ∆*ispA* (NUS3691) were grown in BHI at 37°C in 5% CO_2_ without Zn^2+^ supplementation. Cultures were normalized by their optical density, and diluted into BHI at 37°C with (**A**) or without (**B**) added Zn^2+^. Growth was monitored by measuring optical density over time. Growth curves in (**C**) and (**D**) were performed as above using strain NUS0267 [∆*cpsE* // P_Zn_-*cpsE*] and its derivatives with ∆*cpsC* (NUS0679), ∆*walK* (NUS3688), ∆*cpsC ∆walK* (NUS3698), ∆*ispA* (NUS3708), and ∆*cpsC* ∆*ispA* (NUS3701). Plotted are the means and standard deviations from three biological repeats.

**Fig. S4. The growth defects associated with ∆*cpsA* ∆*walK* cannot be alleviated by inactivating *tacL* or *whyD*.** (**A**) In *S. pneumoniae*, the biosynthesis of WTA and LTA involves the same Und-PP-linked TA precursor, which is made in the cytoplasm, polymerized, and flipped to the outer leaflet of the cell membrane^75^. TacL attaches the glycan moiety of the TA precursor to DAG to produce LTA^34^. Without it, TA precursors are redirected to the WTA pathway^49^. To make WTA, LytR/Psr ligates the TA precursor to the C6 position of MurNAc^34^. Thus, they compete with the PG O-acetylase OatA for the same attachment site. PG can also be modified by PgdA, which removes the acetate group from GlcNAc. PG modifications by OatA and PgdA could influence the activity of PG hydrolases, such as PcsB. WhyD is a WTA hydrolase^72^. When disrupted, the overall level of WTA increases^72^. (**B**) Strain NUS0267 [∆*cpsE* // P_Zn_-*cpsE*] and its derivatives with the indicated deletions were grown to the mid-exponential phase in BHI medium without the Zn^2+^ inducer, serially diluted, and spotted onto blood agar plates with the indicated concentration of Zn^2+^. Plates were incubated overnight at 37°C in 5% CO_2_ before imaging. The experiments were performed three times with similar results.

**Fig. S5. Capsule production induces cell chaining but is not exacerbated in mutants lacking *cpsA* and *walK*.** Flow cytometry experiments were conducted to evaluate chaining in cells when capsule is produced. (**A**) Strain NUS0267 [∆*cpsE* // P_Zn_-*cpsE*] and its derivatives lacking *cpsA* (NUS0796), *walK* (NUS3688), and both (NUS3698) were grown in BHI at 37°C with 5% CO_2_ without added Zn^2+^. Cultures were normalized by optical density and diluted into BHI with or without the indicated amount of Zn^2+^ and incubated for five generations at 37°C with 5% CO_2_. Next, cells were fixed with 1% paraformaldehyde and analyzed by flow cytometry. Cells that form chains cause an increase in side-scattering (SSC) and forward-scattering (FSC). Events were gated based on comparison with the area where ~90% of the parent strain falls when grown without added Zn^2+^ (top left). Shown are representative graphs comparing the SSC area and FSC area from three biological repeats. (**B**) The data from three biological repeats from (**A**) are summarized as a dot plot. Shown are the means and standard deviations. P-values were calculated by one-way ANOVA followed by Tukey’s multiple comparisons test. ****, p < 0.0001; ***, p < 0.001; *, p < 0.05; ns, p > 0.05. (**C**) Strains IU1781 [wild-type; D39W] and HMS0002 [D39W ∆*cpsE*] were grown in the presence or absence of 0.45 mM or 0.85 mM Zn^2+^ until the exponential phase and analyzed by flow cytometry as above. Events were gated against NUS0267 grown without Zn^2+^ as in (**A**). Shown are the means with standard deviations from three biological repeats. P-values were calculated by one-way ANOVA followed by Tukey’s multiple comparisons test. **, p < 0.01; ns, not significant.

**Fig. S6. The chaining phenotype of ∆*walK* is alleviated by inactivating *oatA* or overexpressing *lytR*.** Representative graphs from three biological repeats comparing side-scatter area (SSC-A) and forward-scatter area (FSC-A) of the data summarized in **Fig. 6**, as assessed via flow cytometry. Strain NUS0267 [∆*cpsE* // P_Zn_-*cpsE*] and its derivatives with the indicated modifications were grown in BHI without the Zn^2+^ inducer at 37°C in 5% CO_2_ until cultures reached the early log phase. Cells were collected, inactivated with 1% paraformaldehyde, and analyzed by flow cytometry. Events were gated as described in the legends of **Fig. S4**.

**Fig. S7. Mut-seq identifies residues critical for CpsA function.** (**A**) Strains NUS3688 [∆*cpsE* ∆*walK* // P_Zn_-*cpsE*], NUS3698 [∆*cpsE* ∆*walK* ∆*cpsA* // P_Zn_-*cpsE*], and derivatives of NUS6142 [∆*cpsE* ∆*walK* ∆*cpsA*::P-*sacB-kan-rpsL* // P_Zn_-*cpsE*] were transformed with the indicated *cpsA* alleles and grown in BHI until the exponential phase in the absence of added Zn^2+^. Cultures were serially diluted and spotted on blood agar plates with or without 0.45 mM Zn^2+^ supplementation. Plates were incubated overnight at 37°C in 5% CO_2_ before imaging. The experiments were performed three times with similar results. (**B**) The structural model of CpsA (UniProtKB accession: Q9ZII9) was generated using AlphaFold2 and viewed using PyMOL (<http://pymol.org>). ConSurf was used to colour the model based on sequence conservation (left). Residues identified to be important for function in (**A**) are highlighted as orange sticks (right).

**Fig. S8. Cells ligating WTA exclusively by CpsA formed chains.** (**A**) Strains HMS0002 [∆*cpsE*], NUS7288 [∆*cpsE* ∆*lcp* // P-*cpsA*], and NUS7289 [∆*cpsE* ∆*lcp* // P-*lytR*] were grown in BHI with or without 1 mM IPTG at 37°C in 5% CO_2_ until the early exponential phase. Cells were collected, fixed with 1% paraformaldehyde, and analyzed by flow cytometry. Shown are representative graphs comparing side-scatter area (SSC-A) and forward-scatter area (FSC-A) from three biological repeats. Events were gated against HMS0002 grown without IPTG (bottom left). (**B**) Quantification of data described in (**A**). Shown are the means with standard deviations from three biological repeats. P-values were calculated by one-way ANOVA followed by Tukey’s multiple comparisons test. ****, p < 0.0001; *, p < 0.05; ns, not significant.
